# Sex differences in life-course social disadvantage and all-cause mortality: evidence from a longitudinal study from 1996 to 2023

**DOI:** 10.1186/s12889-026-28773-4

**Published:** 2026-08-03

**Authors:** Daniel Tarekegn Worede, Alfons Hollederer

**Affiliations:** Faculty of Human Science, University of Kassel, Arnold-Bode-Str 10, Kassel, 34127 Germany

**Keywords:** Social Disadvantage, Life Course, SOEP, Sex Differences, Mortality, Germany

## Abstract

**Background:**

Social inequalities in mortality persist in Germany despite high universal health coverage. Yet no study has simultaneously examined sex-specific differences in accumulation, age modification, and life-stage variation in the association between social disadvantage and all-cause mortality across the adult life-course.

**Methods:**

We conducted a longitudinal life-course study using 28 years of data from the German Socioeconomic Panel (SOEP; 1996–2023), comprising 112,006 adults and 660,480 person-wave observations. Low socioeconomic status (low-SES), an indicator of social disadvantage, was measured as the number of years spent in the lowest 20% of a composite SES index of education, occupation, and income. Cox proportional hazards models were fitted to estimate (1) accumulation, (2) age-modification, and (3) life-stage variation in the social disadvantage-mortality association for women and men. Results are reported as adjusted hazard ratios (HRs) with 95% confidence intervals (CIs).

**Results:**

Over 684,295 person-years of follow-up, 4,974 deaths occurred. Cumulative social disadvantage was associated with higher mortality risk for both sexes. Among women, each additional year spent in social disadvantage at age 50 was associated with a 13% higher mortality hazard (HR = 1.13; 95% CI: 1.07–1.19). The accumulation gradient was steeper among men (15%; interaction HR = 1.02; 95% CI: 1.01–1.04). The age-modification model indicated that the strength of the social disadvantage–mortality association attenuated with age, but more gradually for men than for women. In the life-stage model, stronger associations were observed in emerging adulthood for women (ages 16–29: HR = 1.34; 95% CI: 1.05–1.71) and in mid-adulthood (ages 45–64: HR = 1.13; 95% CI: 1 .03–1.24) and late adulthood ( ages 65 + : HR = 1.14; 95%CI: 1.00–1.29) among men.

**Conclusion/outlook:**

An accumulation of social disadvantage increases mortality hazard for both sexes, but is higher for men. Association decreases with age, strongest in emerging adulthood for women and mid-to-late adulthood for men. These findings underscore the importance of life-course–informed and gender-responsive public health actions to reduce persistent social inequalities in mortality in Germany.

**Supplementary Information:**

The online version contains supplementary material available at 10.1186/s12889-026-28773-4.

## Introduction

Social inequalities in morbidity and mortality have long been central to public health research and policy [1–3]. Socioeconomic status (SES) is an indicator of social position within the social hierarchy and a key social determinant of health [4–7]. A substantial body of research has documented that individuals with low-SES (hereafter referred to as “social disadvantage” [8] have higher mortality and morbidity than their more advantaged counterparts [9–11]. For example, a multicohort life-course study in the United States reported a 1.28-fold higher mortality risk among adults of social disadvantage, and a 1.25-fold higher hazard among young adult women [12]. In Germany, data from the Gutenberg Health Cohort Study showed that social disadvantage was associated with a 1.86-fold higher risk of all-cause mortality [10].

Mortality also varies by sex. Globally, men have a 1.6-times higher mortality risk than women [13]. Mortality differences might stem from behavioural, sociocultural and biological variations, including differential exposure to occupational hazards, gendered behavioural norms, and sex-specific hormonal regulation and disease susceptibility [14]. Men more often engage in high-risk behaviours like tobacco and heavy alcohol use, and hazardous risks, reinforced by cultural expectations of masculinity [15]. Women, by contrast, experience higher rates of physical inactivity and obesity, influenced by gender norms [14]. Such patterns underscore the need to examine social disadvantage and mortality through a sex specific lens.

Furthermore, socioeconomic trajectories differ by sex. Men and women differ in labour-market (employment) participation [16], occupational risk [17, 18], and caregiving and domestic roles [19, 20]. These structural differences may influence the accumulation and timing of social disadvantage for women and men. Furthermore, from an intersectional perspective, biological processes, as well as gendered social roles and cultural norms, would be potential sources of differences in social disadvantage and mortality [21]. Yet, despite strong theoretical foundations, empirical evidence on sex differences in the accumulation of social disadvantage across the adult life-course and their mortality associations remains understudied, including in Germany [22].

Age further modifies the association between socioeconomic status and mortality. The two competing hypotheses, “cumulative advantage” versus “age as leveller,” are essential for understanding this Association [23–25]. The former proposes that disparities widen with age, while the latter suggests convergence. Whether these patterns differ by sex and whether the association varies across adult life-stages remains insufficiently investigated, particularly in Germany.

Life-course epidemiology provides a strong framework for studying these gaps. The accumulation model posits that repeated or persistent exposure to social disadvantage exerts a cumulative psychosocial, material and biological toll on health. The critical and sensitive period model suggests that disadvantage during specific developmental or life-stages may be disproportionately associated with later health outcomes, including mortality [26–28].

These theoretical frameworks are well-suited to the public health policy priorities of the WHO and the European Union, which call for robust evidence and the implementation of life-course strategies to achieve universal health coverage and health equity within the commitment of Sustainable Development Goals (SDGs)[29–31]. In addition, scholars underscore that advancing health inequality science requires integrating the life-course perspective into etiologic and intervention public health research, as the interaction between socioeconomic status and health develops over time [32, 33]. Examining the extent to which, for whom, and when social disadvantage is most detrimental is crucial for designing targeted, equitable public health actions.

Germany provides a good context for such analysis, as evidence shows persistent, and in some cases widening, social inequalities in mortality despite high universal health coverage, strong social security systems, and its status as the wealthiest country [1]. Prior evidence consistently shows declines in absolute mortality,however, persistent relative social inequalities in health and mortality have been documented [1, 34, 35]. Nevertheless, the findings remain fragmented due to heterogeneous approaches. For instance, many studies rely on area-level deprivation or administrative data. Earlier SOEP analyses had short follow-ups, used single indicators, a few deaths, and ended in the mid-2010s. Furthermore, they often compared only low versus high SES, failing to capture persistent/repeated accumulated disadvantage [1, 36–38]. Research using a life-course approach remains scarce, yet this approach is relevant for understanding how accumulations of interacting social exposures shape health over time [38, 39]. Notably, the German Socioeconomic Panel (SOEP) provides rich longitudinal data suitable for life-course epidemiological research [40, 41]. Still, few studies have examined sex differences in the accumulation of social disadvantage and their associations with all-cause mortality across the adult life-course over extended periods. This study addresses these gaps using 28 years of SOEP longitudinal data.

This study aims to:

1. Examine sex differences in the accumulation of social disadvantage and estimate its association with all-cause mortality (accumulation model).
2. Investigate whether the association between social disadvantage and mortality changes with age, and whether these age-related patterns differ between women and men (age-modification model).
3. Examine life-stage differences in the association between social disadvantage and mortality for women and men (Life-stage model).

Using life-course models, this study provides granular evidence. It adds new scholarship on how social disadvantage becomes embodied over life courses among women and men in association with all-cause mortality. The findings offer actionable insights for public health actions that inform targeted, life-course-oriented interventions.

## Methods

### Study design and data source

We conducted a longitudinal life-course analysis using data from the SOEP-CORE. The Socioeconomic Panel (SOEP) is an annual panel study of private households in Germany that began in 1984 in the Western and 1990 in the Eastern parts of the country, and provides detailed socioeconomic, demographic, and health data [41, 42]. The study uses a multistage stratified sampling design. It includes regular refreshment samples to maintain representativeness. For this analysis, we used wave 40 (v40.1), with harmonized variables, which provides high-quality longitudinal data [41, 43].

### Study population

The sample included all individuals aged 16 years or older who were interviewed between 1996 and 2023 and had valid data on education, occupation, and household income. The SOEP handles missing values for relevant variables, such as household income, using standard multiple imputation; we used these imputed data. We also handled missing values for educational status using multiple imputation. We included 112,006 individuals who contributed 660,480 person-wave observations to this analysis. Participants entered the risk set at their first eligible interview. They were followed until death, loss to follow-up, or administrative censoring in 2023.

### Outcome

All-cause mortality during the study, coded as 1 for deaths and 0 for those still alive at the end of the study. Information on deaths came from the SOEP survey records using the year of death.

### Exposure

The primary exposure variable was low socioeconomic status (low-SES), derived from a socioeconomic status (SES) composite index based on education, occupation, and net monthly household income. The SES index used: (1) education, measured by the Comparative Analysis of Social Mobility in Industrial Nations (CASMIN); (2) occupational status, using the International Socioeconomic Index of Occupational Status (ISEI) [44],and (3) net equivalised household income, which adjusts for household size and composition, which is calculated after taxes and social security contributions. The net income calculation uses the Organisation for Economic Co-operation and Development (OECD) equivalence scale: 1.0 for the household head, 0.5 for each additional person aged 15 or older, and 0.3 for each child under 15. Total household income was divided by these weights (e.g., 1.0 for a single adult, 1.5 for two adults, 2.1 for two adults with two children), yielding an equivalised income that reflects shared resources and differing needs. Each SES component was classified as low, medium, or high, then scored from 1 to 7, yielding a total SES sum score range of 3 to 21. The total SES sum scores were then grouped as low-SES (the lowest 20%), middle-SES (the middle 60%), or high-SES (the highest 20%) [6, 45],Wulkotte & [46].

Cumulative low socioe-conomic status (cum low-SES) was operationalized as an annually updated cumulative measure of the total number of years an individual had spent in the lowest SES quintile during follow-up. We defined low SES as the lowest quintile of the SES distribution, following the operationalization used in German health inequality research to denote socially disadvantaged groups [1, 6]. At each annual observation, the cumulative measure reflected only the socioe-conomic exposure accumulated up to that point in follow-up and was updated prospectively. All years spent in low SES were accumulated regardless of whether they occurred consecutively, thereby capturing the duration of socioeconomic disadvantage across adulthood [21].

For descriptive analysis, exposure duration was categorized as: a) never (0 years, always middle-SES or high-SES), b) short-exposure (1–5 years), c) medium-exposure (6–10 years), or d) long-exposure (11–28 years) for descriptive analysis (Supplementary Table S1A-E).

### Covariates

Life-course analysis was conducted for sex (male, female) and age. Age was treated as an underlying time scale, and we adapted four age groups as adult life-stages: emerging adulthood (16–29), young adulthood (30–44), mid-adulthood (45–64), and late/older adulthood (65 +)[47]. Models were adjusted for migration background (migrant, non-migrant), marital status (married, separated/divorced, widowed, single) and cohorts (1920–1939, 1940–1954, 1955–1969, and 1970–1984). Sensitivity analyses included self-rated health (SRH) and overall life satisfaction (LS) as potential mediators rather than confounders. These two variables have been collected annually since the beginning of the study. SES shapes both variables and lies on the causal pathway linking long-term social disadvantage to mortality. Adjusting for these variables in the sensitivity model therefore provides an estimate of the extent to which health and well-being attenuate the association between cumulative low-SES-related mortality differences [38, 45, 46].

### Statistical analysis

This study estimated the relative risk of all-cause mortality using a hazard ratio (HR)***.*** Analyses were conducted in Stata/SE 18 (StataCorp). Descriptive statistics summarised sample characteristics, exposure distributions, and crude mortality rates. We assessed unadjusted mortality gradients using Kaplan–Meier survival curves. These were stratified by sex, age group, and duration of cumulative low-SES exposure.

### Survival modelling

We used Stata’s stcox command to fit Cox proportional hazards models [48]. Age is centred at 50 (age = 0) as a reference. Younger ages were negative,older ages were positive. Age as a timescale accounted for age-related mortality hazard [49, 50]. We used delayed entry (left truncation) to ensure that risk sets were correctly defined [51]. Cluster-robust standard errors accounted for repeated observations within individuals. HR and 95% confidence intervals (CIs) were used to report mortality hazard. P < 0.05 is considered significant.

#### Model 1: accumulation model (Aim 1)

First, we tested whether more time in social disadvantage (cumulative low-SES, cum low-SES) environments was associated with a mortality hazard, as an accumulation of risk in Model 1A. Second, to test whether the association differed by sex, we included a sex × cum low-SES interaction term in Model 1B [21, 22, 52]. We used time-varying coefficients to model social disadvantage, since it violated the PH assumption.

#### Model 2: age modification model (Aim 2)

We extended Model 1 by modelling cum low-SES as a time-varying coefficient (tvc), alongside sex and migration background (tvc) (Model 2 A)*.*In this model, we tested whether the association between social disadvantage and mortality changes with age and whether this association differs between men and women. We evaluated sex differences by adding sex as a moderator through cum low-SES × sex (Model 2B). This allowed the association to vary with age and evaluated whether the cum low-SES–mortality association changed continuously as individuals age [21].

#### Model 3: life-stage model (Aim 3)

We extended Model 2 by adding cum low-SES × age-group interaction terms to test whether the SES–mortality association differed across life stages. To examine sex differences, we estimated two models: a main model without the cum low-SES × sex interaction (Model 3 A) and an interaction model including this term (Model 3B). Sex and migration background were modelled as time-varying covariates because they violated the PH assumption. In addition, we conducted sex-stratified analyses (Model 3 C) to examine further whether the life-stage pattern differed between men and women.

#### Model 4: robustness and sensitivity analysis

After running the models, we re-estimated them by adjusting for health and well-being (i.e., SRH & LS). We also did sex-stratified analyses to check differences by sex. We tested the proportional hazards assumption (PH) using Schoenfeld residuals (global goodness-of-fit). We considered it to meet the assumption when P > = 0.05, and we assessed the significance of the interaction term using a likelihood-ratio test in Stata (lrtest).

## Results

### Descriptive characteristics and cumulative low-SES patterns

A total of 112,006 individuals (57,018 women and 54,988 men) were included in the analytical sample, contributing 660,480 person-wave observations. These contributed 684,295 person-years of follow-up, and 4,974 (4.44%) deaths occurred during follow-up time (Supplementary Table S1).

80% of observations had not experienced social disadvantage (are either middle or high SES), while 14% had 1–5 years, 3.8% had 6–10 years, and 2.2% had 11–28 years of exposure. Notably, men were more prevalent in the medium- and long-term exposure groups, whereas women were more prevalent in the short-term exposure group (*Supplementary Table S2*).

This distribution of exposure duration was associated with mortality outcomes. In overall mortality, men accounted for 2,749 (55.3%), while women accounted for the remaining 2,221 (44.7%). Examining sex differences in mortality rates revealed a steeper gradient for men (8.6 to 21.0 per 1,000) than for women (6.5 to 11.9 per 1,000), showing a clear difference between the sexes (*Supplementary Table S3*).

Additionally, exposure duration varied across life-stages: short-term exposure was higher among younger ages, while medium- and long-term exposure was higher in mid- and late adulthood. Notably, long-term exposure was most frequent among those aged 45–64 and 65 +, reflecting accumulation of social disadvantage.

**Fig. 1:**
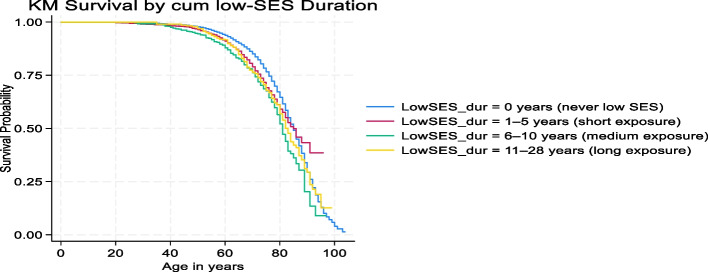
Kaplan–Meier survival by duration of cumulative low-SES exposure

**Fig. 2:**
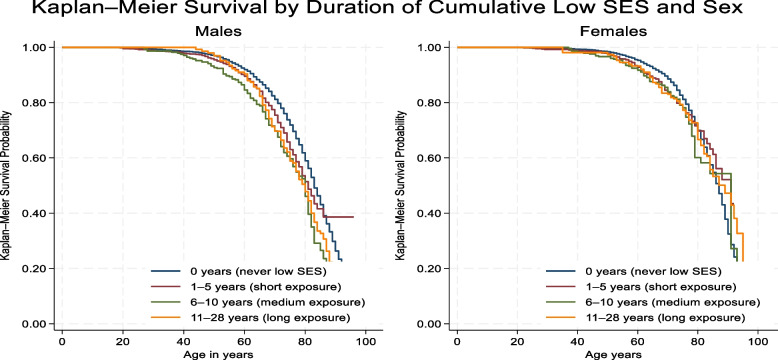
Sex stratified Kaplan–Meier survival by duration of cumulative social disadvantage exposure

Unadjusted gradient patterns were visualized using Kaplan–Meier curves, and we observed differences in survival probability by duration of exposure to social disadvantage (Fig. 1). As exposure duration to social disadvantage increases, the survival probability decreases. A similar monotonic gradient pattern was observed in the sex-stratified Kaplan–Meier curves (Fig. 2). However, because the number of individuals (not observations) with over 11 years of exposure to the accumulation of social disadvantage was very small, the corresponding survival estimates were unstable.

Furthermore, there was a strong, monotonic accumulation of social disadvantage among men, whereas among women it was weaker and less linear, particularly as individuals aged. Notably, the medium and long-term exposure groups showed partial convergence at older ages.

### Cox regression model: analysis results

#### Model 1: accumulation model (aim 1)

At age 50, cumulative exposure to social disadvantage was strongly associated with higher all-cause mortality. For women in the reference group, each additional year spent in low-SES was associated with a 13% higher hazard of mortality (HR = 1.13; 95% CI: 1.07–1.19). Men had higher baseline mortality (HR = 1.61; 95% CI: 1.51–1.72). The significant low-SES × sex interaction (HR = 1.02; 95% CI: 1.01–1.04) indicates that men showed a steeper gradient for social disadvantage. That is, the hazard of social disadvantage for men is 15% per additional year of low-SES (combining the main and interaction effects). The time-varying coefficient for cum low-SES (HR_tvc = 0.998; 95% CI: 0.998–0.999) showed a modest attenuation of the low-SES–mortality association with age (Tables 1 and 2).

**Table 1:** Accumulation (Model 1): Association of social disadvantage and All-Cause Mortality

Model 1A			Model 1B	
Variable	HR 95%CI	*P*-value	HR (95% CI	*P*-value
Cum low-SES	1.15(1.10–1.21)	< 0.001	1.13 (1.07–1.19)	< 0.001
Male (ref. Female)	1.66(1.56–1.77)	< 0.001	1.61 (1.51–1.72)	< 0.001
Male × cum low-SES	–-	––	1.02(1.01–1.04)	0.004
Birth cohort (ref. 1920–1939)				
1940–1954	0.45 (0.41–0.49)	< 0.001	0.45(0.42–0.49)	< 0.001
1955–1969	0.24 (0.20–0.28)	< 0.001	0.24(0.21–0.28)	< 0.001
1970–1984	0.13 (0.10–0.18)	< 0.001	0.13 (0.10–0.18)	< 0.001
Marital status (ref. Married))				
Separated/divorced	1.45 (1.31–1.59)	< 0.001	1.45(1.32–1.60)	< 0.001
Widowed	1.39 (1.28–1.51)	< 0.001	1.39 (1.28–1.51)	< 0.001
single	1.63 (1.43–1.86)	< 0.001	1.62 (1.43–1.86)	< 0.001
Migrant (ref. non-mig)	1.10 (1.01–1.20)	0.036	1.10(1.0032–1.20)	0.042
Time-varying effect				
tvc (cum_lowSES × time)	0.998(0.997–0.999)	< 0.001	0.998(0.997–0.999)	< 0.001

**Table 2:** Association of Sex-specific social disadvantage and All-Cause Mortality

Sex	HR *	95%CI*	P-value
Female (ref)	1.13	1.07–1.19	< 0.001
male	1.15	1.10–1.21	< 0.001

^*^ (linear combination of main and interaction model estimated using lincom, efom Stata command from Table 1)

Cohort variation was observed in the association. Younger cohorts had much lower mortality hazards than the oldest. Marital status was strongly associated with mortality: separation or divorce (HR = 1.45), widowhood (HR = 1.39), or being single (HR = 1.63) increased hazard versus marriage. The association with a migration background is weaker (HR = 1.09) (Table 1).

After adjusting for baseline health and well-being in the sensitivity analysis, the association between accumulation of social disadvantage and mortality weakened but remained significant among women (HR = 1.09; 95% CI: 1.04–1.14). We also conducted a lagged-sensitivity analysis and found similar results. Therefore, sensitivity analyses support the robustness of the findings (Supplementary Tables S4 and S5).

Together, our results support the accumulation model of life-course epidemiology: persistent social disadvantage increases the mortality hazard. Men and women experience social disadvantage differently: men showed a steeper gradient and higher mortality risk than women.

### Model 2. age modification effect (aim 2)

The results showed that social disadvantage remained significantly associated with mortality. Examining age-modification effects, the time-varying coefficient for cum low-SES (HR_tvc = 0.9984; 95% CI: 0.9977–0.9989) indicated that the social disadvantage–mortality association gradually weakened with age (both Models 1 and 2). Additionally, time-varying effects for sex (HR_tvc = 1.007; 95% CI: 1.004–1.012) indicated that the mortality gap between men and women widened with age. Men showed a higher mortality risk than women as they aged. At the same time, the migrant mortality advantage diminished (tvc = 1.009; 95% CI: 1.001–1.012) (Table 3*, Models 2 A and 2B*).

**Table 3:** Age-Modification (Model 2): Time-varying cum Low-SES and All-Cause Mortality

Model 2A			Model 2B	
Variable	HR 95%CI	P-value	HR (95% CI	P-value
Cum low-SES	1.16(1.11–1.22)	0.000	1.14 (1.08–1.19)	0.000
Male (ref. Female)	2.86(2.10–3.91)	0.000	2.67 (1.95–3.66)	0.000
Male × cum low-SES	–-	––	1.03(1.01–1.04)	0.001
Marital status (ref. Married))				
Separated/divorced	1.32 (1.20–1.45)	0.000	1.31(1.21–1.45)	0.000
Widowed	1.41 (1.31–1.52)	0.000	1.41 (1.31–1.52)	0.000
single	1.51(1.33–1.71)	0.000	1.51 (1.33–1.70)	0.000
Migrant (ref. non-mig)	0.57 (0.39–0.85)	0.005	0.57(0.39–0.85)	0.006
Time-varying effect				
tvc(cum_lowSES)	0.998(0.9975–0.9987)	0.000	0.998(0.997–0.999)	0.000
tvc(sex)	1.008(1.004–1.012)	0.00	1.007(1.004–1.012)	0.000
tvc(migrant)	1.009(1.003–1.014)	0.002	1.009 (1.001–1.012)	0.002

Finally, with further adjustment in the sensitivity analysis model for baseline health and life satisfaction, the association of social disadvantage and mortality was attenuated (HR = 1.09; 95% CI: 1.03–1.14), and the cum low-SES × sex interaction was reduced (HR = 1.02; p = 0.046). However, age-modification patterns persisted. Notably, poor self-rated health and lower overall life satisfaction were significant and attenuated the association between social disadvantage and mortality (*Supplementary Table S5*).

#### Model 3. life-stage model (aim 3)

In the main model, social disadvantage was strongly associated with all-cause mortality in emerging adulthood (reference age group: 16–29 years). Among women, each additional year spent in low-SES was associated with a 23% increase in the hazard of mortality (HR = 1.23; 95% CI: 1.08–1.40) (*Model 3A*).

Men had a substantially higher baseline mortality (HR = 2.67; 95% CI: 1.96–3.66). Beyond this baseline difference, the cum low-SES–mortality gradient was steeper among men, as indicated by a significant cum low-SES × sex interaction (HR = 1.02; 95% CI: 1.01–1.03). This corresponds to a 21% increase in the mortality hazard per additional year of low-SES (1.19 × 1.02 ≈ 1.21) (Table 4*, model 3B*).

**Table 4:** Life-Stage Model (Model 3): Age‑Group–Specific Association of Cum Low-SES with All‑Cause Mortality

Model 3A			Model 3B	
Variable	HR (95%CI	*P*-value	HR (95% CI	*P*-value
Cum low-SES (16–29)	1.23 (1.08–1.40)	0.002	1.20 (1.05–1.37)	0.006
Male (ref. Female)	2.85 (2.08–3.89)	< 0.001	2.67 (1.96–3.66)	< 0.001
Sex × cum low-SES (1) male	–-	–-	1.03 (1.01–1.04)	< 0.001
Age‑group interactions (ref: 16–29)				
30–44 × cum low-SES	–-	–-	0.88 (0.76–1.01)	0.074
45–64 × cum low-SES	–-	–-	0.85 (0.75–0.97)	0.018
65 + × cum low-SES	–-	–-	0.83 (0.73–0.95)	0.004
Marital status (ref: Married))				
Separated/divorced	1.32 (1.21–1.45)	< 0.001	1.23 (1.12–1.35)	< 0.001
Widowed	1.42 (1.31–1.53)	< 0.001	1.33 (1.23–1.43)	< 0.001
Single	1.50 (1.33–1.70)	< 0.001	1.42 (1.25–1.60)	< 0.001
Time-varying effects				
tvc(sex)	1.008 (1.003–1.014)	0.002	1.006 (1.001–1.012)	0.037
tvc(migrant)	1.009 (1.003–1.014)	0.002	1.006 (1.001–1.012)	0.032

Age-group–specific cum low-SES association (combined HRs): Multiplying the main cum low-SES hazard (1.20) by each interaction term provides the cum low-SES hazard per age group: 16–29 (HR 1.20), 30–44 (HR 1.06), 45–64 (HR 1.02), and 65 + (HR 0.99) (*Supplementary Table S6*). These results show that early adulthood social disadvantage is strongly associated with mortality. Even without combining the HRs, the results indicate a decrease in the hazard rate with increasing age (Table 4*, Models 3 A and 3B*).

Most importantly, sex-stratified analyses revealed variation in the association across life-stages. For men, social disadvantage was associated with all-cause mortality during young adulthood to late-adulthood life-stages, with a relatively increasing HR observed in mid-adulthood (45–64) (HR 1.13) and late adulthood (65 +) (HR 1.14). For women, it was associated with the emerging and young adulthood life stages, but relatively higher HR was observed in emerging adulthood (18–29) (HR = 1.34) and then declined across life-stages. In general, the association increased for men and decreased for women (Table 5*, Model 3C*).

**Table 5:** Sex-Stratified Analysis of the Association of Cum Low-SES with All‑Cause Mortality (Life-Stage model 3)

Model 3C				
Men (n = 54,988); deaths = 2,749)			Women (*n* = 57,018; deaths = 2,225)	
Variable	HR (95%CI)	*P*-value	HR (95% CI)	*P*-value
Cum low-SES x age group				
16–29 (ref)	1.23 (0.97–1.57)	0.088	1.34 (1.05–1.71)	0.019
30–44	1.11 (1.01–1.22)	0.032	1.15 (1.01–1.30)	0.032
45–64	1.13 (1.03–1.24)	0.011	1.12 (0.99–1.26)	0.075
65 +	1.14 (1.00–1.29)	0.047	1.08 (0.92–1.27)	0.365
Birth cohort (ref: 1920–1939)				
1940–1954	0.48 (0.44–0.54)	< 0.001	0.42 (0.36–0.48)	< 0.001
1955–1969	0.24 (0.20–0.29)	< 0.001	0.28 (0.22–0.35)	< 0.001
1970–1984	0.12 (0.08–0.17)	< 0.001	0.18 (0.12–0.27)	< 0.001
Migback (non-migrant)	1.06 (0.94–1.19)	0.363	1.16 (1.01–1.33)	0.038
Time-varying effects				
tvc(cum low-SES)	0.9987 (0.9971–1.0003)	0.116	0.9990 (0.9970–1.0010)	0.336

migback = migration background, cum low-SES = cumulative low socioeconomic status, tvc time vary effects, Ref = reference category

In the Sensitivity analysis, after adjusting for SRH and LS, the association between social disadvantage and all-cause mortality remained significant across life-stages, showing a gradient pattern, with a relatively higher HR in early life-stages and a lower HR in later life-stages. The cum low-SES × sex interaction remained significant, though smaller in magnitude (HR = 1.02; p = 0.032) (*Supplementary Table S7*).

## Discussion

### Summary of key findings

To our knowledge, this is the first comprehensive epidemiological study using nearly three decades of high-quality SOEP data and a life-course epidemiological approach to investigate sex differences in life-course social disadvantage and all-cause mortality in Germany. The study found a significant association between social disadvantage and all-cause mortality, consistent with existing studies. However, this study extends evidence in three ways. First, repeated and cumulative social disadvantage across the adult life-course matters more than single episodes. Each episode of disadvantage increased the hazard of all-cause mortality for both sexes, but the gradient is steeper in men. Second, as people age, the association becomes weakened, but men’s mortality hazard attenuates more gradually than women’s. Third, the association between social disadvantage and all-cause mortality varied across adult life-stages. For both sexes, social disadvantage was significantly associated with mortality in young adulthood (30–44 years). Among women, this association was also observed during emerging adulthood (16–29 years), whereas among men it extended into mid-adulthood (45–64 years) and late-adulthood (65 +).

### Accumulation of social disadvantage and all-cause mortality

We found that accumulation of social disadvantage is associated with a higher all-cause mortality hazard for both sexes, with a steeper gradient in men (13% per episode in women, 15% in men), supporting the accumulation hypothesis. This finding is consistent with evidence in Germany, which documented income-related mortality gaps:13% of women and 27% of men in the lowest income group die before age 65[1]. However, the existing study measured only income, comparing low- and high-income groups, rather than accounting for repeated and cumulative social disadvantage over time, using time-varying effects and interaction models.

The Gutenberg Health Study similarly found that social disadvantage doubled the risk of mortality and cardiovascular disease [10]. Studies of area deprivation also revealed that residents in disadvantaged areas face higher premature death risks: 33% in women and 43% in men, compared with residents in the less deprived areas [53] and Tetzlaff et al. [54] also reported differences across deprived areas [54]. Yet all of these studies used approaches that differed from our focus on the repeated or cumulative nature of social disadvantage. Our findings, therefore, extend the literature by demonstrating that the persistence of disadvantage, rather than isolated episodes, is particularly harmful.

Several mechanisms may explain the observed sex differences. Social, behavioural, and material pathways, including education, income, occupational exposures, and health behaviours, shape long-term mortality risk [14]. For example, behavioural and material factors could partly explain the association: lower education is associated with lower income and more unfavourable health behaviours.

In contrast, better-educated individuals get better jobs and earn more, which helps reduce mortality hazard [32]. Persistent sex differences in the association might stem from exposure duration, timing, and extent, including occupational hazards, material deprivation, bio-behavioural differences, and gendered norms [14]. Men more often engage in high-risk behaviours, including tobacco, high alcohol use, and hazardous risks, often reinforced by cultural expectations of masculinity, which may strongly affect later-life mortality risk [15].

International studies also support these findings, showing higher mortality risk with increasing social disadvantage. For example, in Singapore, mortality hazard increased with the accumulation of social disadvantages [55]. Likewise, a study analyzing the Wisconsin Longitudinal Study in the United States reported similar results [21].

### Age-modification effects in the SES–mortality association

The association between social disadvantage and mortality weakens with age, consistent with evidence in Germany showing higher mortality inequalities among adults under 50 [1], and declining educational health disparities across older age groups [56]. This pattern aligns with the “age-as-leveller” hypothesis, which posits that selective survival, social protections, and biological ageing reduce relative SES differences later in life.

Age captures both biological processes and cohort membership, meaning that observed differences may reflect biological ageing (ageing effect) as well as generational experiences (cohort effect), as we consider cohorts (as seen in model 1). Although cross-sectional studies cannot infer this evidence [25], our longitudinal design allows us to observe age-related changes (ageing effects) over time, even if we cannot fully disentangle ageing effects from cohort effects.

Comparable findings are reported internationally, with longitudinal studies showing that childhood and adult disadvantage increase mortality hazard [57],[58].

However, disparities exist in international studies also; for example, Pudrovska [22] found in the US that social inequalities widen with age, with the mortality gap increasing by about 10% for women and 8.8% for men each year [22]. Differences across countries, such as welfare systems, pension structures, and healthcare access, likely explain these disparities. Germany’s universal health coverage and strong social security protection may buffer late-life SES disparities, contributing to the attenuation observed in our study.

### Life-stage patterns in the SES–mortality association

Our life-stage model showed different patterns for women and men in the association between social disadvantage and mortality. For women, social disadvantage during emerging adulthood (16–29) and young adulthood (30–44) is associated with later mortality. For men, the association extends into mid-adulthood (45–64) and late adulthood (65 +). Young adulthood showed a significant association for both sexes and might be considered a sensitive period in the life course.

Life-course theory suggests that early adulthood is a sensitive period marked by transitions into completing education, entering the labour market, independent living, and family formation [27],[26],[47]. Disadvantage during this life-stage can shape long-term opportunities, health behaviours, and material conditions. Evidence from the United States shows that persistent neighbourhood disadvantage in young and middle adulthood predicts premature mortality among women [12], and early-life SES has lasting effects on mortality, particularly for women [21]. Our findings suggest that early-to-mid adulthood life-stages may be a sensitive period in the SES–mortality association.

Our findings indicate that the age-modification model shows a general weakening of the association between social disadvantage and all-cause mortality with increasing age. In contrast, the life-stage model reveals that men showed a significant association in mid-to-late adulthood, while women showed a significant association in emerging and young adulthood. This divergence suggests that, while some population-level gains level the gradient, men’s accumulated social disadvantage remains strongly associated during this stage, likely amplified by occupational risks and health behaviours.

Furthermore, the lower mortality risk observed among migrants is consistent with the well-documented “healthy migrant effect,” in which migrants tend to be healthier than the native-born population [59]. However, the diminishing advantage with age suggests convergence over time, potentially due to cumulative exposure to social disadvantage.

Finally, baseline health and well-being partially attenuated the SES–mortality association. These variables occupy a dual role: they are both potential confounders (shaping subsequent SES trajectories) and mediators (reflecting earlier disadvantage). Adjusting for them, therefore, risks over-adjustment. The attenuation observed should thus be interpreted as reflecting both confounding control and partial mediation, rather than pure bias reduction. Our sensitivity analyses speak to robustness rather than causal decomposition.

### Strengths and limitations

This study offers several notable strengths. The use of nearly three decades of high-quality SOEP extended follow-up data enables analysis of exposure accumulation, age-modification effects, and life stages for both women and men. Annual SES measures enable us to construct cumulative low-SES exposure indices that reflect the duration and persistence of social disadvantage across the adult life course. This approach is limited in public health research in Germany. By applying life-course epidemiological models, time-varying coefficients, and interaction terms within Cox models, we provide granular evidence on sex-specific patterns of social disadvantage and mortality.

The study has several limitations. First, some confounding is possible. SOEP does not have consistent and sufficient early-life SES information for most respondents, specifically for the mortality outcome. This limits our ability to model full life-course trajectories from childhood onward. Our estimates, therefore, reflect socioeconomic conditions only from adolescence onward or in adulthood. This may underestimate the total cumulative burden of disadvantage. Second, survey-based mortality data present challenges. SOEP mortality data are typically lower than official German statistical reports, since household members or relatives voluntarily report deaths. This can result in underascertainment and conservative absolute mortality estimates. However, the available evidence indicates that missing deaths are not systematically distributed across SES groups. Therefore, any bias introduced is unlikely to affect the relative estimates of cumulative social disadvantage-mortality associations, central to this study.

Third, the absence of cause-specific mortality data in SOEP prevents us from investigating whether cumulative social disadvantage associations differ for cardiovascular, cancer, or other causes of death.

## Conclusion, policy, and research implications

This study found sex differences in the accumulation of social disadvantage in all-cause mortality inequalities in Germany across the adult life-course. Social disadvantage is associated with an increased hazard of all-cause mortality for both sexes, but is relatively higher for men. The association also decreased with age, being stronger in the emerging-to-young-adult life-stages for women and in the young-to-late-adulthood life-stages for men.

The policy implications. Our findings highlight the importance of considering life-course- and gender-responsive public health actions. Reducing socioeconomic inequalities during emerging and young adulthood could reduce the hazard of all-cause mortality for both women and men. Considering public health strategies, such as the WHO and EU life-course and health-in-all-policies approaches, could help narrow health gaps among women and men, as well as across generations.

Future research should further investigate causal links. Advancing health equity sciences requires leveraging longitudinal data and using advanced epidemiological methods. Examples include causal mediation analysis methods, which can provide causal evidence. These methods could enable granular evidence for monitoring health inequalities. Linking SOEP and cause-of-death registries allows detailed analysis of sex-specific pathways.

To enhance mortality analysis and health equity, we recommend: 1) enhancing SOEP mortality data, 2) linking SOEP with other healthcare datasets, and 3) establishing a national mortality register with information on the social conditions of the deceased. These steps are crucial for monitoring and reducing health inequalities across the life-course in Germany and beyond.

## Supplementary Information

Supplementary Material 1.

## Data Availability

The SOEP is available for non-commercial research under strict data protection. This study uses fully anonymized secondary data collected with informed consent and in accordance with German data protection law. German Institute for Economic Research (DIW Berlin) granted data access under a Scientific Use File contract (registration number 7310). The German Council for Social and Economic Data (RatSWD) has approved the SOEP study, and it is conducted in accordance with the Declaration of Helsinki. All participants provided informed consent at the time of primary data collection. Because the present study uses fully anonymized secondary data, no additional ethical approval was required. Data can be accessed: https://www.diw.de/en/diw_01.c.678568.en/research_data_center_soep.html. As all analyses use anonymized secondary data and no new data is collected, additional ethical approval is not required.
