# Supplementary material for: Sex differences in life-course social disadvantage and all-cause mortality: evidence from a longitudinal study from 1996 to 2023
Source: BMC Public Health. 2026 Aug 3;26:2255. doi: 10.1186/s12889-026-28773-4 (PMC13435383; doi:10.1186/s12889-026-28773-4)
Supplement: Supplementary file 1 [file 12889_2026_28773_MOESM1_ESM.zip › Supplmentary material-p1-Revised.docx]

**Sex Differences in Life-Course Social Disadvantage and All‑Cause Mortality: Evidence from a Longitudinal Study from 1996 to 2023**

**Supplementary file**

**Table of contents**

| Description | Page |
| --- | --- |
| 1. Method | 1 |
| **Supplementary Table S1 A to E**: Construction of SES using Education, Occupational status and net monthly household income | 1 |
| 2. Result | 2 |
| Table S2 frequency of low- SES exposure for 28 years ( separate Excel Table ) | …. |
| Table S3: Descriptive characteristics of cum low-SES exposure and mortality | 2 |
| Table S4: Sensitivity analysis for Model 1 adjusted with SRH and LS | 3 |
| Table S5: Lagged-Sensitivity analysis for Model 1 (accumulation), the association between cum low-SES and mortality | 3 |
| Table S6. Sensitivity analysis for Model 2 adjusted with SRH and LS | 3 |
| Table S7: Age‑Group–Specific Effects of Cumulative Low SES (from lincom, eform Stata command) | 5 |
| Table S8: Sensitivity analysis for Model 3 adjusted with SRH and LS | 5 |

1. **Method section**

**Exposure Variable: SES**

**Supplementary Table S1 A to E: Construction of SES using Education, Occupational status and net monthly household income**

The SES construction follows the original Lamperts et al. (2013) classification, and it is as follows.

A. EDUCATION

| Education level CASMIN | Description | SES point |
| --- | --- | --- |
| 1a,1b | No formal education/primary | 1 |
| 1c | Lower secondary | 2 |
| 2a | intermediate secondary | 3 |
| 2b | General maturity certificate (Abitur) | 4 |
| 2c | Vocational quaölification | 5 |
| 3a | Higher education /technical college | 6 |
| 3b | University degree | 7 |

B. **Occupation: First, we harmonised ISEI88 (1996-2017) and ISEI08 (2018-2023) (Ganzenboom, 1992)**

| Occupational status ISEI88 &ISEI08 | SES point |
| --- | --- |
| no occupation | 1 |
| 11.56-25 | 2 |
| 26-35 | 3 |
| 36-45 | 4 |
| 46-55 | 5 |
| 56-65 | 6 |
| 66-90 | 7 |

C. **Net household (HH) Income**: The 3^rd^ important factor, income, is also classified into 7 categories based on the median national income distribution, following the Lampert et al. (2013) classification for comparability, reproduction, future studies, monitoring, and reporting. We exclude extreme values.

| Income Percentile | SES point |
| --- | --- |
| 1-9 | 1 |
| 10-20 | 2 |
| 21-40 | 3 |
| 41-60 | 4 |
| 60-80 | 5 |
| 81-90 | 6 |
| >90 | 7 |

D. **Summed SES**

Using the summation of education, occupational status and net monthly HH income.

**SES=Education+ Occupation+ Income**: Then the indicators are scored from **3 to 21**, and this score is classified into three major SES indices by putting together

E. **Quantiles**

Robert Kock Institute Income defines five equally sized income groupings (20% each), standardised into five quantiles (Q1-Q5) (Lampert et al.,2013)

| Quantiles | SES point |
| --- | --- |
| 1^st^ (lowest) | 3.0-7.7 |
| 2^nd^ | 7.8-9.6 |
| 3^rd^ | 9.7-11.7 |
| 4^th^ | 11.8-14.1 |
| 5^th^ (Highest) | 14.2-21.0 |

**SES-Index as Categorical:** Using the total SES score, the distribution is therefore divided into:

1. Low SES=1^st^ quantile =20% Low SES = 3.0-7.7. This category is an indicator of the risk of poverty ( Social disadvantage).
2. Middle SES= (2^nd^ 3^rd^ 4^th^ quantiles^)^ =60%	Middle SES=7.8-14.1
3. High SES = (5^th^ Quantile) =20%	High SES=14.2-21.0
2. **Result Section**

**Table S3**. Descriptive characteristics of cumulative low-SES exposure and mortality among 57,018 women and 54,988 men (N = 112,006; deaths = 4,974)

| Exposure | Observation n (%) | Mortality n (%) | | Mortality rate per 1000 person-years | |
| --- | --- | --- | --- | --- | --- |
| Low-SES |  | Men | Women | Men: (95% CI) | Women: (95% CI) |
| 0 (never low-SES) | 528,105 (79.96) | 2,144 (43.1) | 1919 (38.6) | 8.56 (8.20–8.93) | 6.47 (6.19–6.77) |
| 1-5 years | 93,466 (14.15) | 286 (5.7) | 150 (3.1) | 5.29 (4.71–5.94) | 3.47 (2.95–4.07) |
| 6-10 years | 24,735 (3.75) | 166 (3.3) | 71 (1.4) | 12.29(10.55–14.31) | 5.98 (4.74–7.55) |
| 11-28 years | 14,174 (2.15) | 153(3.1) | 85(1.7) | 21.04(17.95–24.65) | 11.86(9.59–14.67) |
| Total | 660, 480 | 2,749 (55.3) | 2,225(44.7) | -- | -- |

**Robustness and Sensitivity Analysis Model 1:** To further examine the robustness of our findings, sensitivity analyses were conducted that adjusted for baseline health status and life satisfaction.

Model 1 (accumulation): The association between social disadvantage and mortality was weakened but remained statistically significant among women (HR = 1.09; 95% CI: 1.04–1.14). This indicates that sex differences in the social disadvantage-mortality gradient were reduced after adjusting for baseline health and well-being. The poor self-reported health has almost tripled the risk of mortality hazard compared to those with good health. Similarly, overall life satisfaction was associated with exposure and outcome, indicating that high (66%) and medium (49%) levels had lower hazard rates than low (33%). These two factors partially attenuated the association between social disadvantage and all-cause mortality (Table S2). Furthermore, we conducted a lagged sensitivity analysis to better account for the possible influence of health deterioration on both SES and mortality. We found that health deterioration does not influence the association between SES and mortality (Table S5).

Table S4: Sensitivity analysis for Model 1 (accumulation), the association between cum low-SES and mortality

| Model 1 | | |
| --- | --- | --- |
| Variable | HR 95%CI | P-value |
| Cum low-SES | 1.09(1.04–1.14) | <0.001 |
| Male (ref. Female) | 1.79(1.68–1.90) | <0.001 |
| 1940-1954 | 0.52 (0.48–0.56) | <0.001 |
| 1955-1969 | 0.29(0.27–0.35) | <0.001 |
| 1970-1984 | 0.18 (0.13–0.24) | <0.001 |
| Separated/ divorced | 1.33 (1.21–1.47) | <0.001 |
| Widowed | 1.30 (1.20–1.41) | <0.001 |
| single | 1.51(1.33–1.73) | <0.001 |
| Migrant (ref. non-mig) | 0.98(0.90–1.08) | 0.076 |
| Poor SRH (ref. good) | 2.97(2.77-3.19) | <0.001 |
| Life satisfaction (ref poor) |  |  |
| Medium | 0.51(0.46-0.55) | <0.001 |
| High | 0.34(0.31-0.37) | <0.001 |
| Tvc( cum_lowSES × time) | 0.9989(0.9982– 0.9996) | <0.001 |

Notes: migback=migration background, cum low-SES = cumulative low socioeconomic status, tvc time vary effects, SRH= self-reported health, LS = overall life satisfaction, Ref = reference category

Table S5: Lagged-Sensitivity analysis for Model 1 (accumulation), the association between cum low-SES and mortality

| *Model 1A* | | | *Model 1B* | |
| --- | --- | --- | --- | --- |
| *Variable* | *HR 95%CI* | *P-value* | *HR (95% CI* | *P-value* |
| *cum_lowSES_lag* | *1.15(1.10–1.22)* | *<0.001* | *1.13 (1.08 – 1.20)* | *<0.001* |
| *Male (ref. Female)* | *1.66(1.56–1.77)* | *<0.001* | *1.61 (1.51 – 1.72)* | *<0.001* |
| *Male × cum_lowSES_lag* | *---* | *----* | *1.02(1.01–1.04)* | *0.005* |
| *Birth cohort (ref. 1920-1939)* | | | | |
| *1940-1954* | *0.45 (0.42–0.49)* | *<0.001* | *0.45(0.42–0.49)* | *<0.001* |
| *1955-1969* | *0.24 (0.21–0.28)* | *<0.001* | *0.24(0.21–0.28)* | *<0.001* |
| *1970-1984* | *0.13 (0.10–0.17)* | *<0.001* | *0.13 (0.10–0.17)* | *<0.001* |
| *Marital status (ref. Married))* | | | | |
| *Separated/ divorced* | *1.45 (1.31–1.59)* | *<0.001* | *1.45(1.32–1.60)* | *<0.001* |
| *Widowed* | *1.39 (1.28–1.51)* | *<0.001* | *1.39 (1.28–1.51)* | *<0.001* |
| *single* | *1.63 (1.43–1.86)* | *<0.001* | *1.63 (1.43–1.86)* | *<0.001* |
| *Migrant (ref. non-mig)* | *1.10 (1.01–1.21)* | *0.026* | *1.10(1.01 – 1.20)* | *0.034* |
| *Time‑varying effect* | | | | |
| *tvc (cum_lowSES_lag × time)* | *0.998(0.997–0.999)* | *<0.001* | *0.998(0.997 – 0.999)* | *<0.001* |

Age modification effect sensitivity analysis: here we also adjusted for SRH and LS; the cum low-SES association was attenuated (HR = 1.09; 95% CI: 1.03–1.14) with the age-like model 1, and the cum low-SES×sex interaction was reduced (HR = 1.02; p = 0.046). However, age‑modification patterns persisted. Notably, poor self‑rated health and lower life satisfaction remained strongly associated with higher mortality, suggesting these factors partially attenuated the cum low-SES-mortality association (Table 3, model 2B).

Table S6. Sensitivity analysis for Age‑Modification Effect (Model 2): Time‑Varying Effects of Low-SES and adjustment with SRH and LS

| Model 2 | | |
| --- | --- | --- |
| Variable | HR 95%CI | P-value |
| Cum low-SES (per year) | 1.09 (1.03–1.14) | <0.001 |
| Male (ref. Female) | 2.27 (1.66–3.10) | <0.001 |
| Male × cum low-SES | 1.02 (1.00–1.03) | 0.046 |
| Separated/ divorced | 1.23 (1.12–1.35) | <0.001 |
| Widowed | 1.33 (1.23–1.43) | <0.001 |
| single | 1.42 (1.25–1.61) | <0.001 |
| Migback (non-migrant) | 0.63 (0.43–0.93) | 0.021 |
| Poor SRH (Ref good) | 2.83 (2.65–3.02) | <0.001 |
| Life satisfaction (ref: low) |  |  |
| Medium | 0.52 (0.48–0.57) | <0.001 |
| high | 0.34 (0.31–0.37) | <0.001 |
| tvc(cum low-SES) | 0.9988(0.9981–0.9994) | <0.001 |
| tvc(sex) | 1.004(1.000–1.008) | <0.061 |
| tvc(migrant) | 1.006(1.001–1.012) | 0.032 |

*Notes: migback=migration background, cum low-SES = cumulative low socioeconomic status, tvc time vary effects, SRH= self-reported health, LS = overall life satisfaction, Ref = reference category*

Table S7: Age‑Group–Specific Effects of Cumulative Low SES (from lincom, eform Stata command)

| Age group | HR | 95% CI | p-value |
| --- | --- | --- | --- |
| 16-29 years | 1.2003 | 1.0531-1.3681 | 0.006 |
| 30-44 years | 1.0571 | 1.0038-1.1131 | 0.035 |
| 45-64 years | 1.0241 | 1.0054-1.0432 | 0.011 |
| 65+ | 0.9923 | 0.9804-1.0045 | 0.215 |

Sensitivity analysis for life stage (model 3): we also adjusted with SRH and LF. The association of social disadvantage in early adulthood remained significant (HR = 1.19; 95% CI: 1.04–1.36). Varied patterns persisted, with the social disadvantage and mortality strongest in early adulthood and weaker in later life. The cumulative low-SES×sex interaction also remained significant but smaller (HR = 1.02; p = 0.032)( Table S7).

Table S8: Sensitivity analysis for Model 3 adjusted with SRH and LS

| *Model 3* | | |
| --- | --- | --- |
| *Variable* | *HR (95%CI* | *P-value* |
| *Cum low-SES (16-29)* | 1.19 (1.04–1.36) | *0.012* |
| *Male (ref. Female)* | 2.26 (1.65–3.09) | *<0.001* |
| *Male × low-SES* | 1.02 (1.00–1.03) | *< 0.001* |
| *30-44* × cum low-SES | 0.88 (0.76–1.01) | 0.073 |
| *45-64* × cum low-SES | 0.85 (0.74–0.97) | 0.017 |
| *65+*× cum low-SES | 0.83 (0.73–0.95) | 0.007 |
| *Separated/ divorced* | 1.23 (1.12–1.35) | *<0.001* |
| *Widowed* | 1.33 (1.23–1.43) | *<0.001* |
| *single* | 1.42 (1.25–1.60) | *<0.001* |
| *Migback (non-migrant)* | 0.64 (0.44–0.94) | *0.024* |
| *tvc(cum low-SES)* | 1.004 (1.000–1.008) | *<0.064* |
| *tvc(sex)* | 1.006 (1.001–1.011) | *0.037* |
| *tvc(migrant)* | *1.006 (1.001–1.012)* | *0.032* |
